# Ferroptosis and tumor immunotherapy: A promising combination therapy for tumors

**DOI:** 10.3389/fonc.2023.1119369

**Published:** 2023-02-08

**Authors:** Huazhong Cai, Yongfei Ren, Shuangwei Chen, Yue Wang, Liangmei Chu

**Affiliations:** ^1^ Department of Emergency, Affiliated Hospital of Jiangsu University, Zhenjiang, China; ^2^ Department of Radiation Oncology, Institute of Oncology, Affiliated Hospital of Jiangsu University, Zhenjiang, China

**Keywords:** ferroptosis, tumor immunotherapy, immune cells, immune checkpoint, T cell

## Abstract

Low response rate and treatment resistance are frequent problems in the immunotherapy of tumors, resulting in the unsatisfactory therapeutic effects. Ferroptosis is a form of cell death characterized by the accumulation of lipid peroxides. In recent years, it has been found that ferroptosis may be related to the treatment of cancer. Various immune cells (including macrophages and CD8^+^ T cells) can induce ferroptosis of tumor cells, and synergistically enhance the anti-tumor immune effects. However, the mechanisms are different for each cell types. DAMP released *in vitro* by cancer cells undergoing ferroptosis lead to the maturation of dendritic cells, cross-induction of CD8^+^ T cells, IFN-γ production and M1 macrophage production. Thus, it activates the adaptability of the tumor microenvironment and forms positive feedback of the immune response. It suggests that induction of ferroptosis may contribute to reducing resistance of cancer immunotherapy and has great potential in cancer therapy. Further research into the link between ferroptosis and tumor immunotherapy may offer hope for those cancers that are difficult to treat. In this review, we focus on the role of ferroptosis in tumor immunotherapy, explore the role of ferroptosis in various immune cells, and discuss potential applications of ferroptosis in tumor immunotherapy.

## Introduction

Cancer immunotherapy includes cancer vaccines, chimeric antigen receptor-T (CAR-T) therapy, cytokine therapy, immune checkpoint inhibitor therapy (ICI), and tumor-targeting monoclonal antibodies (1–3). In the last decade, tumor immunotherapy has flourished, and immunotherapy is considered one of the most promising approaches for systemic cancer therapies, playing an indispensable role in improving therapeutic effectiveness (4). Immune checkpoint inhibitors represented by PD-1/PD-L1 inhibitors that restore T cell anti-tumor activity by blocking the PD1/PD-L1 pathway are more used and effective immunotherapy therapies (5). But the treatment usually benefits only some patients and some types of tumors such as NSCLC, urothelial carcinoma, triple-negative breast cancer (TNBC), and Merkel cell carcinoma (6–9).

Ferroptosis is a novel mode of cell death that refers to iron-dependent regulatory death resulting from lipid peroxidation and consequent cytomembrane rupture. It has received much attention since it was first proposed in 2012 (10). The mechanism is mainly impaired scavenging or overproduction of lipid peroxides, which reaches lethal levels and triggers ferroptosis. Oxidized phosphatidylethanolamine is crucial for the induction of ferroptosis; it can induce ferroptosis by blocking the cystine/glutamate antiporter (system xc^-^) or glutathione peroxidase 4 (GPX4), which leads to defects in the glutathione (GSH) redox system (11). Although previous antitumor treatments have produced remarkable results, tumor recurrences and drug resistance make these tumors difficult to control. Whereas the induction of immunogenic necroptosis of cancer cells in experimental mouse models appears promising in activating antitumor immunity, it is vital to emphasize that several cancers frequently develop necrosis resistance, such as bladder and colorectal cancers (12–14). Hence it is necessary to seek out new manners to lead tumor cells to die in ways besides apoptosis and necrosis. Recent studies have found that ferroptosis, in addition to apoptosis and necrosis, is another choice to overcome cell death resistance and improve the effectiveness of antitumor therapy (15).

## Mechanism and regulation of ferroptosis

### Iron accumulation

It is currently believed that the rapid production of reactive oxygen species (ROS) by cells is the main trigger of ferroptosis (16). Since reactive oxygen species are mainly derived from the Fenton reaction or constitute iron-containing pro-oxidant enzymes, ferroptosis is regulated by pathways related to iron uptake, release, storage, and utilization (17). Iron is mainly transported through FPN or exported by ferritin-containing multivesicular bodies (MVBs) mediated by prominin-2 (prom2) (18). Iron storage in ferritin requires the temporary formation of the GSH-iron complex, so the depletion of GSH promotes the accumulation of unstable iron (19). As we all know, lysosomes are reservoirs of iron with the ability to induce ferroptosis potentially (20). Knockout of lysosomal protein prosaposin mediated the accumulation of lysosomal iron and ROS, causing neuronal ferroptosis (21). In addition, some kinds of brain injuries are also associated with iron overload (22). A model of brain injury was created by injecting FeCl_3_ into the somatosensory cortex of rats. Ferrostatin-1 attenuated both seizures and reduced cognitive function symptoms in these rats, suggesting that this brain injury model may involve ferroptosis (23).

### Lipid oxidation

Ferroptosis depends on disruptions in lipid metabolism; accumulation of lipid oxides and ROS is required. Excessive lipid peroxidation is a hallmark of ferroptosis (24). Polyunsaturated fatty acids (PUFAs) are substrates for lipid peroxidation (25). PUFAs are oxidized by the ACSL4-LPCAT3-lipoxygenase (ALOX) axis (11, 26). It has been suggested that cytochrome P450 oxidoreductase (POR) may promote lipid peroxidation by accelerating the circulation between Fe (II) and Fe (III) in the heme fractions of cytochrome P450 enzymes (CYPs) (27). NADPH oxidase (NOX) uses NADPH as a substrate to transfer electrons to oxygen to generate superoxide radicals, which can promote lipid peroxidation and subsequent ferroptosis (28). The electron acceptor CYB5A is reduced by the electron-accepting NADPH, which in turn triggers lipid peroxidation by extracting methylene hydrogen from PUFAs or reducing iron (15). Arachidonic acid lipoxygenases (ALOXs) are generally thought to cause ferroptosis by catalyzing the accumulation of ferroptosis-associated lipid signals (FALIS), such as oxidized PL-PUFA (26). In addition to the generation of ROS, the mitochondrial respiratory chain also takes part in ferroptosis through lipid oxidation (29). In phospholipid membranes, the peroxidation reaction is passed down (30). SCD1 and ACSL3-mediated monounsaturated fatty acid (MUFA) synthesis inhibit lipid peroxidation (31, 32). Further, transcription factors within the Hippo pathway (i.e., YAP1 and WWTR1) contribute to ferroptosis by modulating the expression of ferroptosis-regulatory genes (e.g., ACSL4, TFRC) (33–35). Several pathways play a crucial role in the determination of ferroptosis susceptibility, covering E-cadherin-NF2-Hippo-YAP/TAZ (34). This is because numerous elements of this pathway are commonly mutated in cancers, and this ferroptosis is additionally discovered in non-epithelial cells that do not express E-calmodulin (15, 36, 37). The mechanism of ferroptosis inhibition may therefore be mediated also by similar calmodulin or cell adhesion molecules. The Hippo-YAP pathway is vital in the development and interacts with other varied signal pathways, thus serving an overwhelming prospective link betwixt normal cell biology and ferroptosis (38).

### Disruption of the antioxidant system

The system xc^–^ GSH- GPX4 pathway is the major anti-ferroptosis network (39). System xc^-^ includes subunits SLC7A11 and SLC3A2 in the cell membrane of the organism. Through the exogenous pathway system xc^-^, cystine and glutamate can be exchanged into the cell in a 1:1 ratio and rapidly reduced to cysteine, thus participating in the cellular synthesis of GSH and GPX4 as free radical scavengers (40, 41). GPX4, a selenoprotein, can produce resistance to ferroptosis by using glutathione as a cofactor and acting as a reductase of peroxides to prevent the buildup of lipid peroxidation and the yield of ROS (17, 42, 43). Since GPX4 is a selenoprotein, the availability of Se also becomes one of the keys to its regulation (44). Due to their general repressive regulation in cells, GPX4 and FSP1 transcriptional, translational, and post-translational levels work upon ferroptosis progression (45–47). Furthermore, glutamate levels also affect the function of system xc^-^, and high extracellular concentrations of glutamate inhibit system xc^-^ and thus eventuate ferroptosis (48). Erastin Prevents cystine uptake by inhibiting System xc-, thereby eliminating GSH. This leads to a decrease in GPX4 activity, which in turn increases the accumulation of ROS, eventually leading to ferroptosis (49). Whereas RSL3 directly inhibits GPX4, driving cell death (50, 51).

### Biomarkers of ferroptosis

Previous studies have shown that ferroptosis causes cell membrane breakage and cell death. During this period, mitochondria become smaller, and membrane density increases. In the nucleus, morphological changes are not obvious, but chromatin agglutination is lacking (10, 52, 53). While this is different from the features of conventional cell apoptosis, such as chromatin condensation, DNA breaks, apoptosome formation, and cytoskeletal disassembly (54). The contents of ACSL4 and TFRC increased during ferroptosis, while the contents of Ferritin, ARNTL, and VDAC2/3 decreased (11, 55, 56). Also, damage-associated molecular pattern molecules (DAMPs) release inflammatory medium (such as high mobility group box 1, etc.) (57). Nuclear factor erythroid 2-related factor 2(NRF2) restricts the occurrence of ferroptosis by transcriptional activation of several cellular protective genes involved in iron metabolism, GSH metabolism, and ROS detoxification enzymes, such as SLC40A1, SLC7A11, and TXNRD1. Thus NRF2 is a key ferroptotic mediator (58). GPX4 and FTH1 are two typical protein markers and protective factors in ferroptosis (10, 59). CHAC1 may be downstream of the GCN2-eIF2α-ATF4 pathway and induce ferroptosis by mediating GSH degradation (60). Expression of PTGS2 (also known as COX2) is increased during ferroptosis (59). Therefore, overexpression of CHAC1 and PTGS2 is also widely considered as biomarkers of ferroptosis. While in apoptosis, the activation of the Caspase system is crucial (61). The increase in Caspase-3 is considered an important marker of apoptosis (62). Moreover, Bax/Bcl-2 has also been shown to be one of the major markers of apoptosis (63, 64).

**Figure 1:**
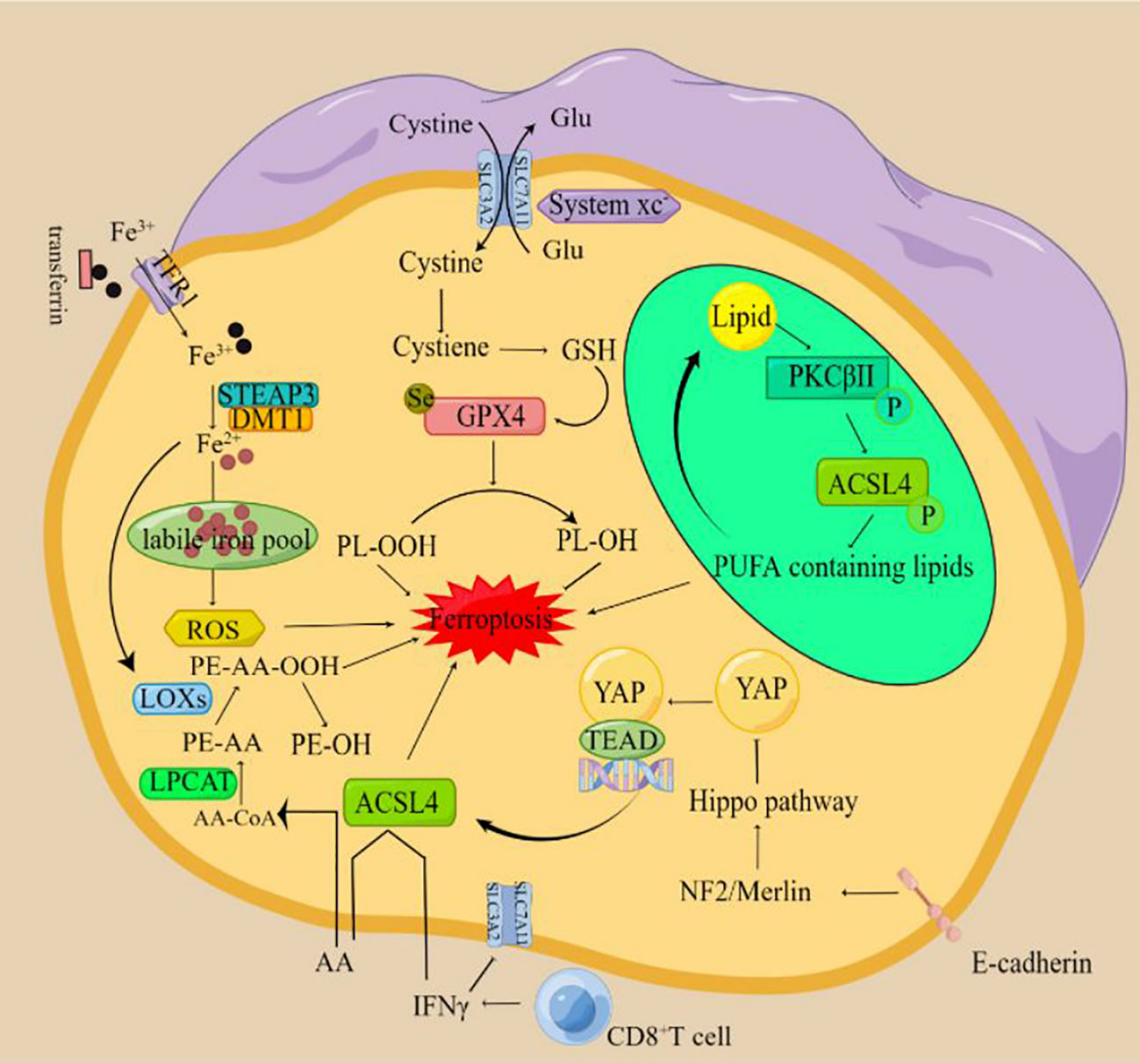
The mechanism of ferroptosis (By Figdraw) The xc-complex introduces cystine into cells by a 1:1 transmigration of glutamate. Once inside the cell, cystine can be oxidized to cysteine, which is used to synthesize glutathione (GSH). Glutathione peroxidase 4 (GPX4) can reduce lipid peroxides to lipid alcohols by using GSH as a reducing cofactor, thus avoiding the occurrence of ferroptosis. Fe^3+^ circulating in the blood enters cells through transferrin receptor (TFR1) -mediated endocytosis and is reduced to Fe^2+^ by six-transmembrane epithelial antigen of the prostate 3 (STEAP3). The divalent metal transporter (DMT1) mediates the release of Fe^2+^ to form the labile iron pool (LIP), resulting in ferroptosis. When cells are treated with a ferroptosis inducer, PKCβII will first sense the increase of lipid peroxidation level in the cell and then be in an activated state. After activation, PKCβII activates ACSL4 through direct phosphorylation of ACSL4 and finally induces a large accumulation of lipid peroxides in the cell and causes ferroptosis. IFN-γ released by CD8^+^ T cells down-regulated the expression of SLC3A2 and SLC7A11, inhibited cystine uptake in tumor cells and promoted lipid peroxidation and ferroptosis in tumor cells. T cell-derived IFN-γ and AA synergistically induced immunogenic ferroptosis in tumor cells through an ACSL4-dependent mechanism. E-cadherin can inhibit the nuclear translocation and transcriptional activity of the transcriptional coregulator YAP by activating Hippo signaling through NF2 (also known as Merlin) tumor suppressor protein. Because YAP targets several regulators of ferroptosis, including ACSL4 and transferrin receptor TfR1, the occurrence of ferroptosis ultimately depends on the activity of the Hippo pathway. Inhibition of the Hippo pathway and activation of YAP can promote ferroptosis.

Currently, the agents that induce ferroptosis are mainly through targeted inhibition of SLC7A11 or GPX4 activity or expression (65–67). Other intracellular anti-ferroptosis systems, including coenzyme Q10 (CoQ10) generated by AIFM2, tetrahydrobiopterin (BH4) generated by GCH1 and ESCRTIII membrane repair system, also play important anti-ferroptosis roles (46, 47, 50, 68, 69). Some important key tumor regulators, RAS, TP53, NRF2, and HIF, are able to regulate the susceptibility of tumor cells to ferroptosis by affecting the metabolism associated with iron, lipids, or ROS (70–74).SLC7A11 is also a biomarker associated with HPV, with low levels of SLC7A11 expression in HPV-positive cancer tissues, and HPV16-derived E6 and E7 proteins inducing EMT activate the expression of transcription factors (e.g., Slug, Twist, ZEB1 and ZEB2) while decreasing GSH levels and sensitizing tumor cells to ferroptosis elicited by erastin (75). In addition, it is particularly noteworthy that tumor cells in the epithelial-mesenchymal transition (EMT) state are particularly sensitive to ferroptosis, which can be associated with the activation of Hippo pathway transcription factors (e.g., YAP and TAZ) (34)(**Figure 1**). These transcription factors promote the expression of key ferroptosis regulators (e.g., ACSL4 and TFRC) (73). A variety of selective autophagy, like ferritinophagy, lipophagy, clockophagy, and molecular chaperone-mediated autophagy (CMA), can stimulate ferroptosis by causing the accumulation of iron and PUFA or protein degradation of GPX4 (56, 76–78).

## Ferroptosis in antitumor immunity

### T cells

**Figure 2:**
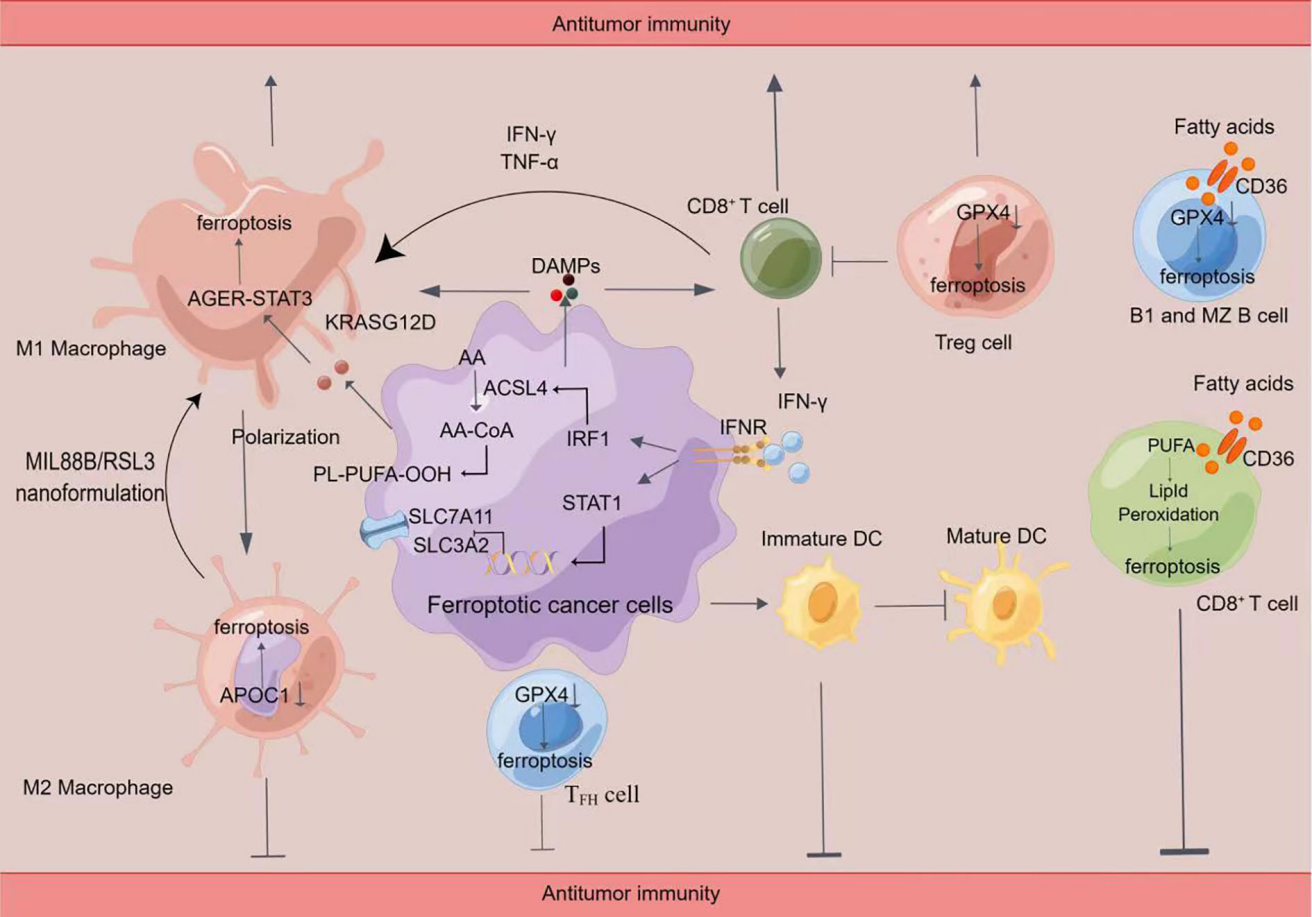
Crosstalk between immune cells and ferroptosis(By Figdraw)Induction of ferroptosis promotes the release of damage-associated molecular patterns (DAMPs), which in turn activates immune cell activity, including CD8^+^ T cells and macrophages. Ferroptosis cells significantly cause M1 polarization in macrophages. Ferroptosis can lead to the release of KRASG12D protein from PDAC cells to promote M2 polarization in macrophages *via* STAT3-dependent fatty acid oxidation. Inhibition of APOC1 or administration of MIL88B/RSL3 nanoformulation promoted the conversion of M2 macrophages into M1 macrophages through ferroptosis. In addition, the release of IFN-γ by CD8^+^ T cells inhibited the xc (-) system (SLC7A11/SLC3A2) and synergistic-induced ferroptosis in tumor cells through ACSL4, leading to increased sensitivity to ferroptosis. Inhibition of GPX4 caused ferroptosis in Treg cells, T_FH_ cells, B1, and MZB cells. Fatty acids in the tumor microenvironment induce ferroptosis in CD8^+^ T cells in a CD36-dependent manner. Then impairing the antitumor function of CD8^+^ T cells. Tumor cells in the early stages of ferroptosis are able to affect the maturation of DC cells and inhibit the phagocytosis of tumor cells by DC cells.

T cells belong to the adaptive immunity of the immune system and have an irreplaceable role in antitumor immunity (79). Zhou X et al. unconcealed that ferroptosis is important in T cell-induced cancer cell death and that HnRNP L promotes cancer immune escape in part by targeting the YY1/programmed death ligand-1(PD-L1) axis and inhibiting ferroptosis in castration-resistant prostate cancer (CRPC) cells. This study suggests that the knockdown of HnRNP L may be a new way to enhance the PD-L1/programmed cell death protein 1(PD-1) blockade strategy of the anti-tumor immune response in CRPC (80). A research team demonstrated that ferroptosis results in the antitumor effects of CD8^+^ T cells and enhance the effectiveness of anti-PD-1/PD-L1 therapy. The investigators found that as CD8^+^ T cells activated by anti-PD-L1 immunotherapy secreted interferon-gamma (IFN-γ) after PD-L1 blockade, IFN-γ significantly downregulates SLC7A11 and SLC3A2 expression in tumor cells through activation of JAK1-STAT1 signaling, leading to reduced cystine uptake, enhanced lipid peroxidation and subsequent ferroptosis (**Figure 2**). Effective antitumor immunity can be generated through the induction of ferroptosis (81). Not coincidentally, it has been shown that CD36 expression is added in tumor-infiltrating CD8^+^ lymphocytes (CD8^+^-TILs). Intrinsic CD36 of T cells promotes oxidized lipid uptake and induces lipid peroxidation, leading to CD8^+^ T cell dysfunction (82, 83). These conclusions reveal ferroptosis of CD8^+^ T cells as a new pattern of tumor immunosuppression and highlight the underlying treatment of blocking CD36 to enhance antitumor immunity. Thus, immune checkpoint blockade (ICB) based immunotherapy could be enhanced by blocking CD36 expression or by adding inhibitors of ferroptosis. This research suggests GPX4 can regulate the antitumor function of CD8^+^ T cells as well (82). Some investigators found that IFN-γ secreted by CD8^+^ T cells redirects lipid metabolism in cancer cells *via* acyl coenzyme, a synthase long-chain family member 4 (ACSL4). ACSL4 activates PUFAs and enhances the sensitivity of tumor cells to ferroptosis in an immunotherapeutically associated setting (84).

Interestingly, in addition to inducing tumorigenic ferroptosis, ferroptosis may also occur in T cells themselves, weakening their immune response (82). Previous studies have found that high levels of ROS may block the differentiation, maturation, and activation of T cells and induce T cell death by enhancing vascular endothelial growth factor (VEGF) production and reducing CD3ζ chains that mediate signaling in T cells (85–87). An increase in VEGF blocks the differentiation and/or migration of thymus-committed progenitors, thereby interfering with T-cell development and leading to tumor-associated immunodeficiency (88). The reduced expression of the CD3ζ chain causes the inhibition of T cell functions, such as proliferative capacity, cytotoxic activity, and cytokine production, ultimately inhibiting the immune response (85, 86, 89).Gpx4-deficient T cells rapidly accumulate membrane lipid peroxides while undergoing ferroptosis (90, 91). Blocking ferroptosis in T cells may then contribute to antitumor immunity. A recent study showed that lipid peroxidation protects cytotoxic T lymphocyte subset 9 (Tc9) cells from tumor or tumor microenvironment (TME) induced lipid peroxidation and ferroptosis through the IL-9/stat3/fatty acid oxidation (FAO) pathway, which is enhanced by STAT3 signaling activation. Regulation of T cell lipid peroxidation could be supplied in the place of augmented T cells-based immunotherapy in human cancers. It suggests that inhibition of ferroptosis or enhancement of FAO may be good manners to transform the anti-tumor efficiency of adoptive cell transfer (ACT)-based immunotherapy. It also emphasizes that targeting lipid metabolism or lipid peroxidation in T cells is worthwhile, which holds promise for improving their clinical efficacy in cancer immunotherapy (92). Notably, the latest research proved that ACSL4-dependent ferroptosis is a new pattern of target cell killing in CD8^+^ T cells (CTL) (93). This discovery not only provides new insights into the mechanism of CTL lethality but also suggests the possibility of an interaction between the two cell death types. However, the molecular mechanism by which AA functions is unclear and remains to be investigated.

Previous evidence suggests that Regulatory T (Treg) cells are important for promoting tumor immune evasion (94, 95). Therefore inhibition of Treg cell’s survival in TME has emerged as a promising research direction in antitumor immunotherapy (96). Gpx4 is essential for shielding activated Treg cells from lipid peroxidation and ferroptosis accumulation and maintaining the Treg cell’s activation and function. It has been shown that Gpx4 deficiency-induced ferroptosis in Treg cells within tumors enhances antitumor immunity and promotes tumor regression (91). It has been recently reported that Gpx4-deficient Treg cells may have a potential role in promoting the activation of Dendritic cells (DC) and the function of CD8^+^ T cells in suppressing tumor growth (97, 98). Thus, studies of ferroptosis-associated Treg cells could serve as a promising method to improve antitumor immunotherapy.

Lipid peroxidation is a previously unrecognized point of mechanical interaction between cancer radiation therapy and immunotherapy. Furthermore, inhibition or resistance to ferroptosis *in vivo* emaciates the synergy between immunotherapy and radiation therapy (99). Major immunosuppressive mechanisms in the tumor microenvironment include high PD-L1 expression and regulatory T cell infiltration (100, 101). Radiation can target PD-L1-expressing cells and regulatory T cells (102, 103). The detection of cGAS in cytoplasmic DNA and micronuclei after radiation has appeared as an important link between natural immunity and radiation therapy as an effective therapeutic strategy for certain cancers (104).

## Macrophages

There are two main subtypes of tumor-associated macrophages (TAMs), namely, antitumor M1-like macrophages and pro-tumor M2 TAMs (105, 106). The number and distribution of TAMs may be associated with the prognosis of underlying malignant disease (107). M1-like macrophages have higher nitric oxide radicals due to higher intracellular inducible NO synthase (iNOS) content than M2-like macrophages, thus inhibiting lipid peroxidation; in contrast, M2-like macrophages have a less inhibitory effect on lipid peroxidation due to lower iNOS content and less production of nitric oxide radicals (108–110). Therefore, RSL3 could not induce ferroptosis in M1-like macrophages but could induce ferroptosis in M2-like macrophages (111). Previous studies showed that increased ROS activated TNF-α/TNFR1 signaling and PI3K/Akt signaling, promoting the secretion of VEGF-C, CCL7, IL-8, and CSF-1, thus leading to macrophage recruitment (112, 113). Some researchers have demonstrated that macrophages and microglia of M1 and M2 macrophages to ferroptosis inducers in mouse models and tumor microenvironments. This suggests that the different sensitivity of macrophages and microglia to ferroptosis in different periods is the different intracellular levels of reactive nitrogen (111). The role of ferroptosis in M1 and M2-like macrophages needs to be further investigated.

Some researchers have demonstrated that oxidative stress can induce the release of KRASG12D protein from PDAC tumor cells leading to autophagy-dependent ferroptosis. They found that KRASG12D protein induces fatty acid oxidation in a STAT3-dependent manner, driving and polarizing macrophages into a pro-tumor TAM similar to M2. Inhibition of KRASG12D release *via* tumor cell exosomes and uptake by immune cells reduces macrophage-induced PDAC tumor growth *in vivo*. Interestingly, KRASG12D enrichment in TAM is related to poor prognosis in PDAC patients, and thus KRASG12D communication from malignant tumor cells to immune cells can be used as a therapeutic target (114). Some investigators demonstrated that the interaction between triple-negative breast cancer (TNBC) cells and TAMs promoted the durative activation of HLF in cancer cells *via* the IL-6-TGF-β1 axis. Next, HLF induces resistance to ferroptosis in TNBC cells through GGT1 and promotes malignancy development. This finding has implications for discovering promising treatment targets for TNBC (115). But studies have demonstrated that curing tumors by ferroptosis could have drawbacks. In pancreatic ductal adenocarcinoma, ferroptosis could lead to experimental pancreatitis and contribute to Kras-driven Pancreatic cancer, which should be considered before using ferroptosis as clinical therapy (116). Recently, some investigators have successfully polarized macrophages from a pro-tumor M2 to an anti-tumor M1 phenotype by ferroptosis using MIL88B/RSL3 nanoformulation (117). This ferroptosis-enhanced macrophage modulation strategy may apply to other combinations of iron-based nanomaterials and iron-related lethal agents.

TAM was discovered to be involved in ferroptosis-mediated immunosuppression (118). Inhibition of ferroptosis associated with PD-1/L1 blockade produced synergistic therapeutic results in the glioblastoma (GBM) mouse model (117). One investigator found that in lung adenocarcinoma patients, RRM2 inhibition promoted M1 polarization and inhibited M2 polarization, which could be inverted by ferroptosis inhibition (119). This suggests that ferroptosis may contribute to the antitumor effects of macrophages. It has been discovered that MIF is highly expressed in nasopharyngeal carcinoma cells, and exosomes secreted by nasopharyngeal carcinoma cells can be ingested by macrophages, thus inhibiting ferroptosis in macrophages and eventually promoting nasopharyngeal carcinoma metastasis. It is suggested that targeting MIF perhaps is a promising therapeutic approach to decrease the metastasis rate (120).

Researchers recently found that asbestos-engulfing macrophages produce ferroptosis-dependent extracellular vesicles (FedEVs) that are taken up by recipient mesothelial cells and produce genomic damage and malignant mesotheliomagenesis (121). This study demonstrates a novel mechanism by which FedEVs contribute to asbestos-induced mesotheliomagenesis by transporting iron as a key mutagenic mediator. A recent study found that CD24^high^ cells are insensitive to paclitaxel but sensitive to ferroptosis agonists. They designed a precision-targeted therapeutic system that can target CD24^high^ cells by enhancing ferroptosis and macrophage phagocytosis through FSP1 and CD24 inhibition mediated by the NF2-YAP signaling axis, finally leading to cell death and hence inhibiting TNBC tumor growth and even disappearance of some tumors (122). This composite nano precision therapeutic system may be a promising means for TNBC therapy. A growing number of researches have indicated that macrophages play a key role in tumor development, metastasis, immune regulation, tumor angiogenesis, TME remodeling, and response to cancer therapy. Macrophage-targeted therapy may be the next frontier in tumor immunotherapy.

## B cells

B cells have both tumor-suppressive and tumor-promoting roles, and the tumor microenvironment can induce B cells to differentiate into different functional subpopulations, thus affecting patient prognosis (123, 124). The balance between tumor suppression and tumor promotion by B cells depends on a variety of factors (123). A recent study found that activation of lipid metabolism by the EBV transformation program generates lipid ROS byproducts to varying degrees and that the Burkitt-like phase of B-cell growth requires detoxification of lipid ROS by GPX4 and its cofactor glutathione. This response, in turn, induces ferroptosis, suggesting that ferroptosis induction may serve as a towardly therapeutic pattern for the prevention or treatment of certain EBV^+^ lymphomas (125). Studies have further found EBV infection has a vital impact on redox homeostasis, revealing the role of GPX4 in tumor progression and providing a latent novel target for the therapy of EBV-related cancers (126).

Different B-cell subsets exhibit different sensitivities to ferroptosis. One study found that Gpx4 was essential for the survival of B1 and marginal zone (MZ) B cells but did not affect the survival of follicular B2 cells. Knockdown of the GPX4 gene in B1 and MZ B cells affected the immune response of B cells by inducing cellular ferroptosis; however, knockdown of GPX4 in follicular B cells (FO B) did not cause ferroptosis in the cells (127, 128). This may be because B1 and MZ B cells express significantly higher levels of CD36, thus, are more susceptible to lipid peroxidation by lipid uptake (128). Excessive mitochondrial reactive oxygen species (mtROS) synthesis may inhibit B cell activation and differentiation into antibody-producing plasmatoblasts (129). Increased mtROS may also downregulate CD19 expression to inhibit antibody production (130). Tumor-infiltrating B cells are chiefly derived from memory B cells and consist of diverse subpopulations with contrary functions in cancer immunity (131). However, to date, no studies have shown a relationship between ferroptosis and tumor-infiltrating B cells. It has been found that T follicular helper cells (TFH), activated by ICB in TNBC, modulate the production of antibodies by B cells, which may be crucial for the efficacy of immunotherapy (132). There is growing evidence that the functional status of tumor-infiltrating B cells depends on whether they are aggregated in structurally well-developed tertiary lymphoid structures (TLS) (131). The development of means to activate B cells, or to promote antibody production, may be useful for the immunotherapy of tumors.

## DCs

Dendritic cells (DCs) play an important role in antitumor immunity by activating cytotoxic T cells as antigen-presenting cells of the immune system (133). Glutathione levels regulate DC differentiation and function as APCs (134). Depletion of GSH leads to severe cellular dysfunction by inhibiting DC maturation and the production of inflammatory cytokines (135). Indirect evidence suggests that ferroptosis also has an impact on DCs function (136). Tumor-associated DCs typically exhibit a reduced ability to process antigens due to elevated lipid levels, a feature that correlates with susceptibility to ferroptosis (74, 77). Recently, it has been demonstrated that increased ferroptosis impairs the maturation of dendritic cells and their function in tumor suppression. Utilizing an immunogenic cell death-based DCs vaccine model, they expanded on that PPARG-mediated ferroptosis of dendritic cells limits anti-tumor immunity in mice (137). Recent studies have found that ferroptosis cancer cells can inhibit the cross-presentation of soluble antigens in DC cells. They found that tumor cells early in ferroptosis were able to affect DC cell maturation and inhibit DC cell phagocytosis of tumor cells. This suggests that ferroptosis negatively impacts the antigen-presenting cells and the adaptive immune response, which might hinder therapeutic applications of ferroptosis induction (136). Previous studies have found that Sesn2, a highly conserved stress-inducible protein, protected DCs from iron death, thus improving the immune function of DCs and response in septic mice (138). These discoveries proved a new role for ferroptosis DCs in driving the immunosuppressive tumor microenvironment.

## Ferroptosis and ICIs

Anti-cancer immunotherapies targeting the programmed cell death/-ligand 1(PD-1/PD-L1) and cytotoxic T lymphocyte antigen 4 (CTLA-4), commonly spoken as immune checkpoint inhibitors (ICIs), have proven to be effective in many tumors (139). In distinction to chemotherapy or targeted therapy, ICIs will induce sturdy immune responses during a bound share of patients, even once therapy stops, showing that ICIs can generate durable tumor-specific immune memory (140–144).

Efimova et al. first demonstrated that ferroptosis is immunogenic *in vitro*/*in vivo* and have shown that ATP and HMGB1 (the most prominent damage-associated molecules) are released passively after the ferroptosis timeline, followed by passive release (12). Since immunogenic molecules are closely associated with the immunogenicity of primitive ferroptosis tumor cells, this implies that ferroptosis is crucial for immunotherapeutic efficacy (12, 73). The combination of immunotherapy with ferroptosis promotes a synergistic effect to inhibit tumor growth. Induction of ferroptosis by direct or indirect means, such as radiation therapy and targeted therapy, emerges as a promising combination modality for improving anti-PD-1/PD-L1 immunotherapy (145). However, drug resistance is also an inescapable and urgent problem. Recently, some researchers have found increased expression of TYR03 in anti-PD-1 resistant tumors. Further studies revealed that the TYR03 signaling pathway upregulates the expression of key ferroptosis genes such as SLC3A2 to inhibit tumorigenic ferroptosis and that resistance to anti-PD-L1 therapy can be overcome by using TYR03 receptor tyrosine kinase (RTK) inhibitors. And this inhibitor can inhibit ferroptosis. Researchers found that inhibition of TYR03 promoted ferroptosis and sensitized tumors to anti-PD-1 therapy in a TNBC homozygous mouse model. This study reveals that disabling ferroptosis through TYR03 inhibitors is an effective strategy for overcoming immunotherapy resistance (146).

Recent studies have found that in GBM, triple-negative breast cancer, and pancreatic tumors, ferroptosis inhibition combined with anti-PD-1 can improve the efficacy of single immunotherapy, suggesting a synergistic effect of ferroptosis inhibition and anti-PD-1 (147–149). When mice were treated with the immunotherapeutic drug immune checkpoint inhibitor in combination with a ferroptosis sensitizer, the effect on tumor growth was significantly stronger than when either drug agent was used alone. The combined use of ferroptosis sensitizers and immune checkpoint inhibitors produced a strong immune response that fought tumors by promoting ferroptosis (150). Therefore, there is a need to further explore the modalities of ferroptosis regulation in combination with immunotherapy and provide new opportunities for future research directions.

## Nanomaterials and small-molecule drugs associated with ferroptosis

The use of nanoparticles to precisely induce ferroptosis is a recent hot research direction. Self-amplifying nanoparticles RCH NPs reprogram the two-sided nature of ferroptosis by amplifying its positive effects while reversing its inherent negative effects to achieve ferroptosis tumor therapy (151). Ultrasmall single-crystal Fe nanoparticles (bcc-USINPs) can be excreted from the kidney without toxic effects on normal organ tissues (152). Platelet membrane-coated magnetic nanoparticles induce the onset of ferroptosis in tumor cells (153). Magnetic nanoparticles can induce ferroptosis in tumor cells by applying external RSL-3 encapsulated nanoparticles that can induce immunogenic death of tumor cells by applying external light (40). Nanoreactors can disrupt the dynamic balance of redox and iron metabolism without relying on the Fenton reaction and thus induce ferroptosis (154). DNA-functionalized self-assembled ferric tetroxide nanosystems that trigger specific ferroptosis/chemotherapy by dual catalysis of ATP and acid and enable precise monitoring of its therapeutic process (CACN). Dynamic nanoparticles activated by an acidic intracellular environment, a nanoplatform for tumor microenvironment-specific triggering of ferroptosis and manganese ion release associated with T1/T2 bimodal high-field magnetic resonance imaging (R-PtWMn) (155). These studies aim to improve the efficacy of tumor immunotherapy by inducing ferroptosis in various ways.

A recent study found a dual PI3K/HDAC inhibitor, BEBT-908, targeting both PI3K and HDAC signaling pathways, which can effectively inhibit tumor growth by promoting cellular ferroptosis and enhance the immunogenicity of tumor cells and enhance the effect of immune checkpoint inhibitors (150). Some investigators have treated metal-polyphenol networks (PFG MPNs) with light. For precision photothermal therapy. PTT or iron ion-mediated ferroptosis in this system enhances the immunogenic death effect and will effectively unlock the inhibitory effect of exosomes on DC maturation (156). Another study recently reported an Ir Rome number 3 containing a complex of ferrocene-modified diphosphine ligands localized to the lysosome (Ir1). In the acidic environment of lysosomes, Ir1 effectively catalyzes Fenton-like reactions that generate hydroxyl radicals, induce lipid peroxidation, downregulate glutathione peroxidase 4, and lead to ferroptosis (157). These nanoscale drugs or complexes may bring benefits to eliminate drug resistance in tumor immunotherapy and are expected to significantly enhance the efficacy of tumor immunotherapy in the future.

In addition to the findings in the article, these discoveries or inventions lately are listed in **Table 1**.

**Table 1:** Recently developed nanoparticles or complexes related to ferroptosis.

Name	Encapsulation	Mechanism	Ref.
RCH NPs	Ferric porphyrin,celecoxib,roscovitine and human serum albumin	Induces the secretion of IFN-γ and inhibits GPX4	(105)
Bcc-USINPs	Fe_3_O_4_ and Fe(0)	Iron ion increasing and promote Fenton reaction	(106)
Fe3O4-SAS@PLT	Fe_3_O_4_,platelet and sulfasalazine	Inhibiting the glutamate-cystine antiporter system X	(107)
Nanoreactor	MnO,glucose oxidase and polyethylene glycol	Consuming glutathione and oxidizing glucose	(108)
Ternary alloy PtWMn nanocube	PtWMn	Consume excess glutathione	(109)
PFG MPNs	Fe^3+^ and exosome inhibitor (GW4869)	Fe^3+^ increasing	(110)
Ir1	Ferrocene	Catalyze Fenton-like reaction, produce hydroxyl radicals, induce lipid peroxidation, down-regulate GPX4	(111)

## Concluding remarks

Ferroptosis combined with immunotherapy has achieved considerable results in recent tumor therapy studies. Many types of research have shown that targeting ferroptosis contributes to antitumor immunity, raising the promise for drug-resistant tumor therapy. The study of nanomaterials combined with ferroptosis has also further promoted the development of ferroptosis in tumor treatment. However, despite promising therapeutic applications for ferroptosis induction, how ferroptosis interacts with the immune system remains unclear. Immune cells in TME may be susceptible to ferroptosis, so it is particularly important to study the specific mechanism of ferroptosis in immune cells. The balance of ferroptosis susceptibility in cancer cells, antitumor immune cells, and immunosuppressive cells is still inadequate and needs further research. Research in recent years has explored the use of ferroptosis inducers (FIN) for cancer treatment or suggested combining FIN with other therapies, such as immunotherapy in cancer treatment. Further exploration of ferroptosis regulation in combination with immunotherapy is the key point of future research. This requires a deeper understanding of the regulatory mechanisms of ferroptosis in the genetic background corresponding to cancer due to the great genetic heterogeneity between different cancers. CAR-T cell therapy has major breakthroughs in the treatment of many relapsed or refractory hematologic tumors, but it has been difficult to achieve breakthrough research results in the treatment of solid tumors. Combining CAR-T cell therapy with ferroptosis may be an opportunity to break through this difficulty. But there are very few studies on this area. In addition, the signaling pathways and major transcriptional regulators of ferroptosis need to be further investigated to better regulate ferroptosis for cancer treatment. We believe that with further research, ferroptosis will hold promise for the therapeutic efficacy of cancer, especially immunotherapy.

## Author contributions

HC, YR, SC, YW and LC collected the related papers and drafted the manuscript. HC initiated the study and revised and finalized the manuscript. All authors contributed to the article and approved the submitted version.
